# Research hotspots and frontiers about role of visual perception in stroke: A bibliometric study

**DOI:** 10.3389/fneur.2022.958875

**Published:** 2022-09-16

**Authors:** Nannan Zhang, Chong Li, Jianmin Chen, Xiahua Liu, Zhiyong Wang, Jun Ni

**Affiliations:** ^1^Department of Rehabilitation Medicine, The First Affiliated Hospital, Fujian Medical University, Fuzhou, China; ^2^School of Kinesiology, Shanghai University of Sport, Shanghai, China

**Keywords:** visual perception, stroke, CiteSpace, bibliometric, visual analysis

## Abstract

**Background:**

Visual perception is a dynamic process of perceiving the environment through sensory input and transforming sensory input into meaningful concepts related to environmental visual knowledge. Many studies focusing on the role of visual perception after stroke have been published in various journals. However, a bibliometric analysis in the domain of visual perception after stroke is still lacking. This study aimed to deliver a visual analysis to analyze the global trends in research on the role of visual perception after stroke in the last 10 years.

**Methods:**

The literature was derived from the Web of Science core collection database from 2012 to 2021. The collected material was limited to English articles and reviews. CiteSpace and Microsoft Excel were used for bibliographic analysis.

**Results:**

A total of 298 articles were included in the analysis. The annual number of publications increased from 23 to 42 in the last decade. Rehabilitation was the main research hotspot (*n* = 85). *Journal of Physical Therapy Science* published the largest number of papers (*n* = 14). The most influential author, institution, and country were Rowe FJ (*n* = 17), League of European Research Universities (*n* = 45), and England (*n* = 54), respectively. The keywords with the longest burst period are *field defect, hemineglect, disorder*, and *quality of life*.

**Conclusion:**

This study analyzes the papers on the role of visual perception after stroke in the past 10 years and provides a new perspective for research in this field. At present, the number of articles in this field is not large and the cooperation network is not close enough. In the future, it is necessary to strengthen the cooperation among various countries, institutions, and authors. In addition, large samples and randomized controlled trials are needed to identify the potential treatments and pathophysiology for visual perceptual impairment after stroke.

## Introduction

Visual perception is a dynamic process of perceiving the environment through sensory input and transforming sensory input into meaningful concepts related to environmental visual knowledge, such as visual reception and visual cognition (decision-based analyses combining prior knowledge with retinal input to generate representations) (1, 2). After the stroke, different visual perception disorders will appear due to injury to different brain regions, such as impaired visual memory, visuospatial disturbances, and agnosia (3). In an acute adult stroke population, Rowe et al. reported that 73% of stroke survivors had visual problems, such as impaired central vision (56%), eye movement abnormalities (40%), visual field loss (28%), visual inattention (27%), and visual perceptual disorders (5%) (4). In addition, Rowe and colleagues reported that 20.5% of stroke survivors with a suspected visual difficulty have visual perception deficits (5). Siong et al. indicated that the percentage of visual problems in Hong Kong post-stroke patients was generally lower than in Western countries. However, a high prevalence of patients had deficits in oculomotor (53.1%) and visual acuity (29.8%) (6). In clinical work, clinicians often underdiagnosed visual perceptual deficits in stroke survivors due to reliance on subjective and non-standardized screening approaches (7). Inadequate diagnosis of vision problems can seriously affect the quality of life, functional outcomes, community participation, and independence of stroke survivors (8–10).

Because the emphasis of clinical work often focuses on limb dysfunction after stroke, the visual perceptual problems of patients are often ignored. With the development of modern medicine, the deficits of visual perception after stroke have recently attracted the attention of clinical work (11). With the deepening of research, many studies have focused on visual perception in stroke, and related articles have been published in academic journals. These studies have studied the visual perception of stroke from the basic, clinical, and rehabilitation (12). However, a quantitative analysis of publications focused on the role of visual perception in stroke has not yet been conducted.

Bibliometric analysis is a quantitative statistical method that uses computer image processing technology to convert data into graphics and display them on the screen. Based on the co-citation analysis theory and pathfinding network algorithm, CiteSpace software can analyze literature of specific disciplines or fields from multiple perspectives and draw visual maps to explore the critical paths, research hotspots, and frontiers of the evolution of this discipline or field (13–15). Studies have shown that the CiteSpace software can help scholars to understand the current situation and hotspots of a certain research field more quickly. At present, visual analysis based on the CiteSpace software has been used in various research fields (16, 17).

To address the weakness of quantitative analysis for studies focused on the role of visual perception after stroke, the objective of this study is to use CiteSpace software to perform bibliometric analysis for the global scientific research on studies involved in the role of visual perception after stroke in the past decade.

## Methods and materials

### Data source and search strategy

This bibliometric analysis included published articles and reviews that focused on the role of visual perception after stroke. The core collection database in the Web of Science (WoS) from 1 January 2012 to 31 December 2021 was used as the source for retrieval. Searches were conducted using the following MeSH terms: (((((TS=(visual perception)) OR TS=(visual cognition)) OR TS=(visual^*^)) OR TS=(vision^*^)) OR TS=(neglect)) AND TS=(stroke).

### Inclusion and exclusion criteria

**Figure 1:**
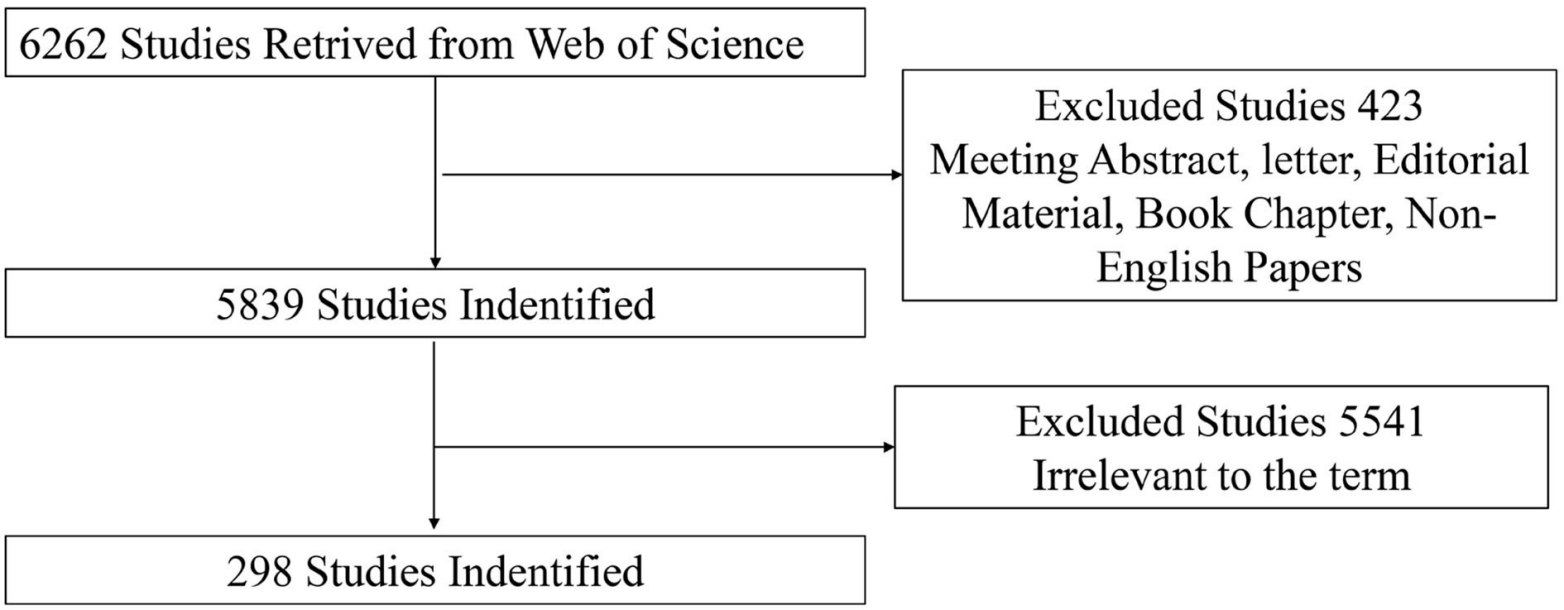
Flowchart of studies inclusion.

The previous studies focused on the role of visual perception after stroke were selected after screening the title and abstract. Only articles and reviews that studied visual perception after stroke were included. Other document types, such as letters, commentaries, and meeting abstracts, were excluded. In addition, the publication language was limited to English. Two researchers independently screened titles and abstracts manually for potential eligibility. Doubtful records were discussed and an independent third reviewer decided whether doubtful records should be included. The flow chart of the inclusion is shown in Figure 1. Finally, 298 records were identified for bibliometric analysis.

### Software parameter settings

CiteSpace V (version: 5.8.R7; Drexel University, USA) and Microsoft Excel 2019 were used for bibliographic analysis. The “Time Sliding” value was set to 1 year. The type of node was selected according to the purpose of the analysis.

### Interpretation of main parameters

#### Node circle and the link between nodes

The radius of a node circle indicates the number of papers published in the author or institutional co-authorship network, and also indicates the frequency of keywords in the co-occurrence network. A link indicates the presence of co-authorship or a co-occurrence relationship.

#### Betweenness centrality

Betweenness centrality is an index that measures the importance of nodes in the network.

#### Burst detection

The function of Burst detection is to detect the situation where there is a great change in the number of citations in a certain period. Thus, it can be used to find the decline or rise of keywords.

#### Dual-map overlaps

Dual-map overlaps are a new method to display the distribution and citation trajectory of papers in various disciplines. As a result, there is a distribution of citing journals on the left side and a distribution of cited journals on the right side. The curve is the citation line, which completely shows the context of the citation.

#### H-index

H-index is the number of publications whose citations are larger than H, which is used to evaluate academic achievements.

## Results

### Publication outputs analysis

**Figure 2:**
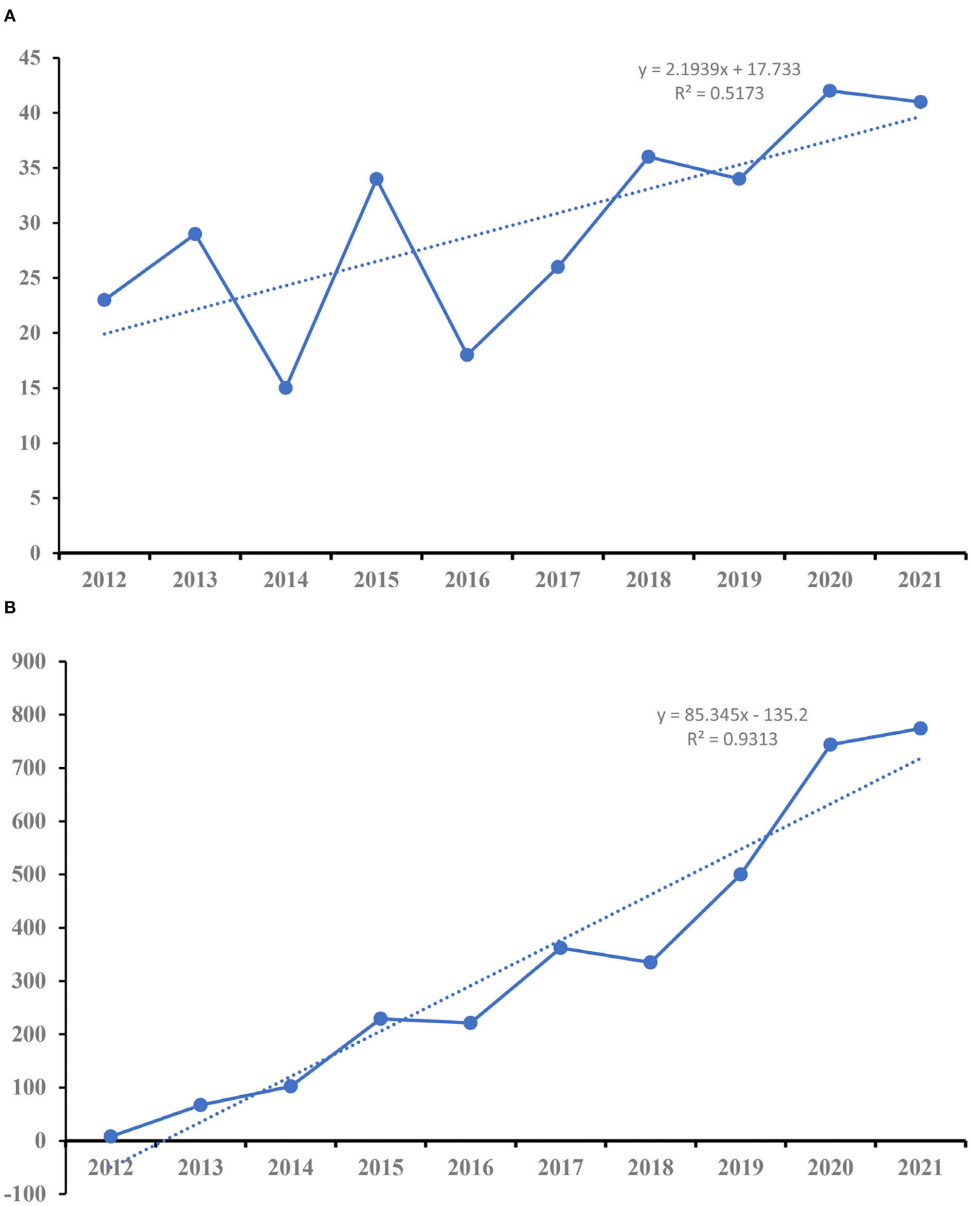
The number of publications and citations. **(A)** The number of annual publications on included studies. **(B)** The number of annual citations on included studies.

A total of 298 publications were included in our study. Figure 2A shows the distribution of annual papers on the role of visual perception after stroke from 2012 to 2021. We use a linear regression equation to fit and analyze the data, and a positive value of x indicates that the data show positive growth. The *R*^2^ represents the degree of correlation, and the larger the number, the stronger the degree of correlation. Two stages have been found in the past decade. The annual literature publication shows an unstable trend from 2012 to 2015, and increases year by year from 2016 to 2021. However, the overall trend shows an upward trend with the increase in the number of years. Figure 2B shows the distribution of annual citations of included studies. The overall trend is positive and the time trend of citations indicated a significant correlation (*R*^2^ = 0.9313).

**Figure 3:**
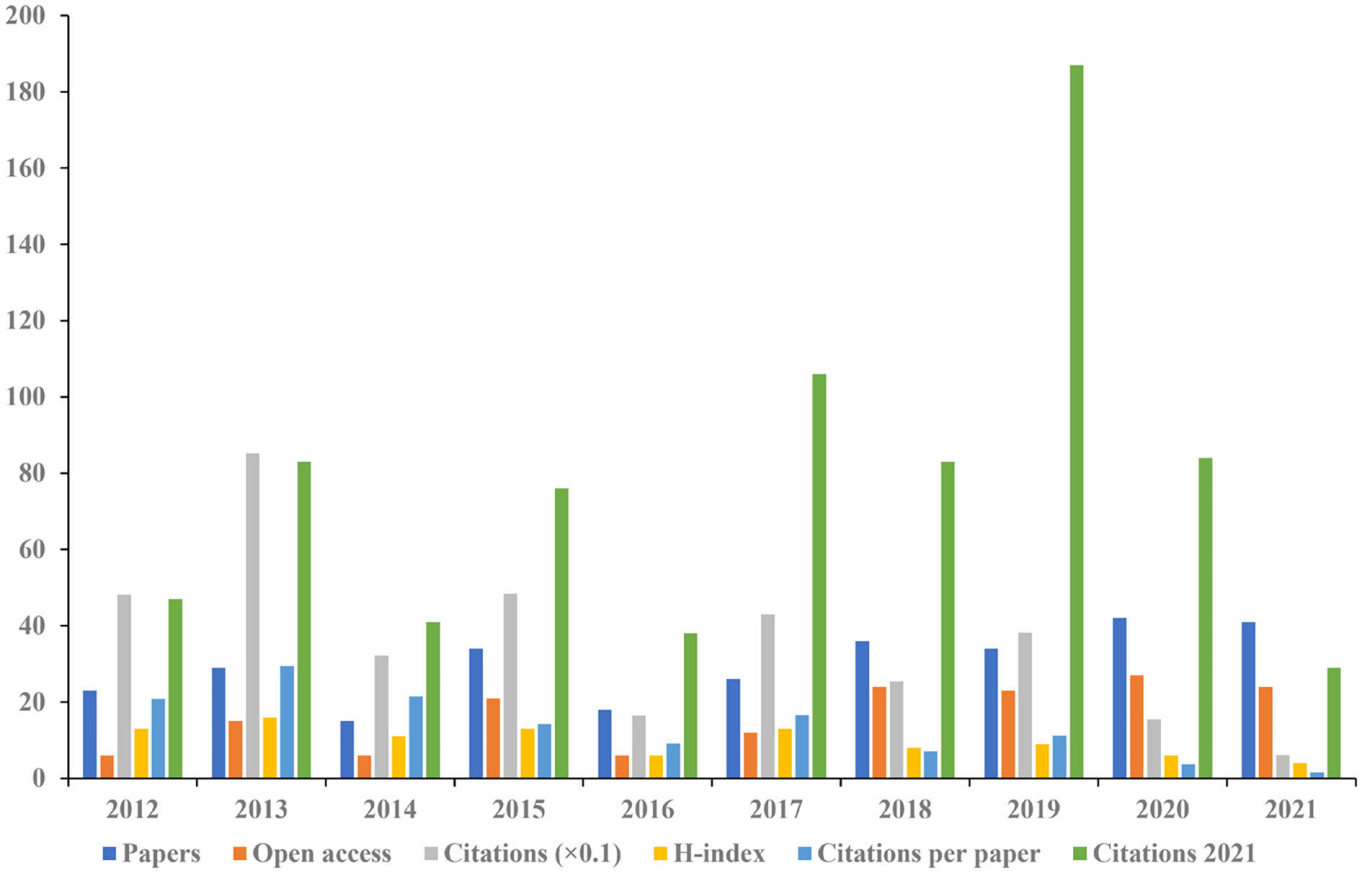
The number of articles, open-access articles, citations, H-index, citations per paper, and citations in 2021 for each year.

As shown in Figure 3, the most published articles (*n* = 42) and open access (*n* = 27) were recorded in 2020. The highest citations (*n* = 852) and H-index (*n* = 16) were occurred in 2013.

### Authoritative journals analysis

A total of 119 journals contributed to the 298 papers included in this study. Table 1 shows the top 15 journals ranked by the number of publications. *Journal of Physical Therapy Science* contributed the most number of publications (*n* = 14), the highest open access (*n* = 13), and the greatest H-index (*n* = 8), followed by *Neurorehabilitation and Neural Repair* (*n* = 12), and *Topics in Stroke Rehabilitation* (*n* = 11). *Neurorehabilitation and Neural Repair* had the greatest citations (*n* = 241). *Annals of Physical and Rehabilitation Medicine* presented with the highest Impact Factor (IF 2020 = 4.919).

**Table 1:** The top 10 journals ranked by the number of publications.

**Journals**	**Papers**	**Citations (WoS)**	**Citations per paper**	**Open access**	**WoS categories**	**IF (2020)**	**Quartile of JCR**	**H-index**
Journal of Physical Therapy Science	14	151	10.79	13	Rehabilitation	/	Q4	8
Neurorehabilitation and Neural Repair	12	241	20.08	5	Clinical Neurology	3.919	Q2	8
Topics in Stroke Rehabilitation	11	78	7.09	3	Clinical Medicine	2.119	Q3	5
Cortex	10	216	21.6	6	Behavioral Sciences; Neurosciences	4.027	Q1; Q2	5
Frontiers in Human Neuroscience	10	136	13.6	10	Neurosciences	3.169	Q3	6
Frontiers in Neurology	10	34	3.4	10	Clinical Neurology; Neurosciences	4.003	Q2; Q2	4
Disability and Rehabilitation	9	38	4.22	4	Social Science	3.033	Q1	3
Neuropsychologia	8	130	16.25	3	Behavioral Sciences; Neurosciences	3.169	Q2; Q3	5
Neuropsychological Rehabilitation	8	101	12.63	4	Neurosciences	2.868	Q3	5
Annals of Physical and Rehabilitation Medicine	7	77	11	5	Clinical Medicine	4.919	Q1	4
Brain and Behavior	7	85	12.14	7	Behavioral Sciences; Neurosciences	2.708	Q3; Q3	5
Journal of Stroke Cerebrovascular Diseases	7	46	6.57	0	Peripheral Vascular Disease; Neurosciences	2.136	Q4; Q4	4
British Journal of Occupational Therapy	6	9	1.5	5	Social Science	1.243	Q4	2
Restorative Neurology and Neuroscience	6	21	3.5	2	Neurosciences	2.406	Q4	3
Plos One	5	126	25.2	5	Multidisciplinary Sciences	3.24	Q2	5

WoS, Web of Science; IF, Impact Factor; JCR, Journal Citation Reports.

**Figure 4:**
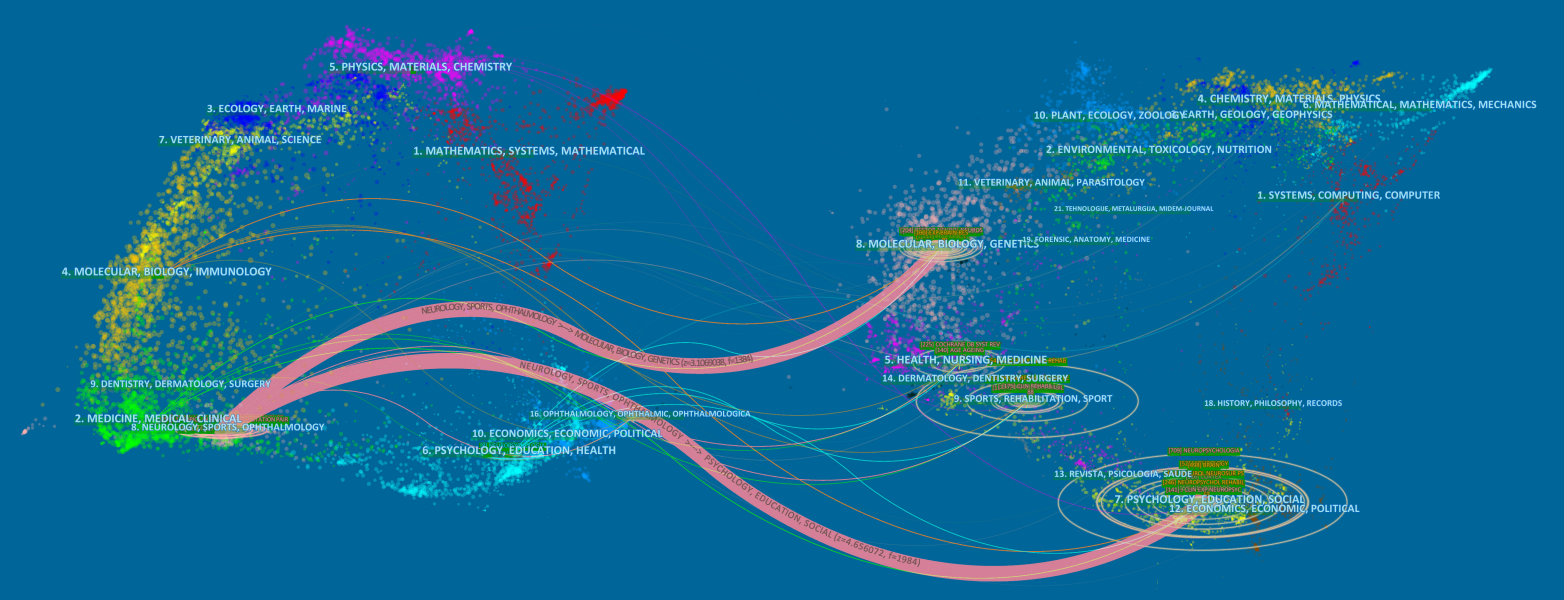
Visualization of dual-map overlays of citing journals and cited journals of 298 studies published from 2012 to 2021. The colored curve indicates the path of citation, which originates from 16 fields of the citing journals on the left and points to 18 fields of the cited journals on the right.

Dual-map overlaps of journals are displayed in Figure 4. The 298 publications included in our study were mostly published in journals dedicated to neurology, sports, and the ophthalmology field. Cited journal publications were mainly from the psychology, education, and social field.

### Subject categories analysis

**Figure 5:**
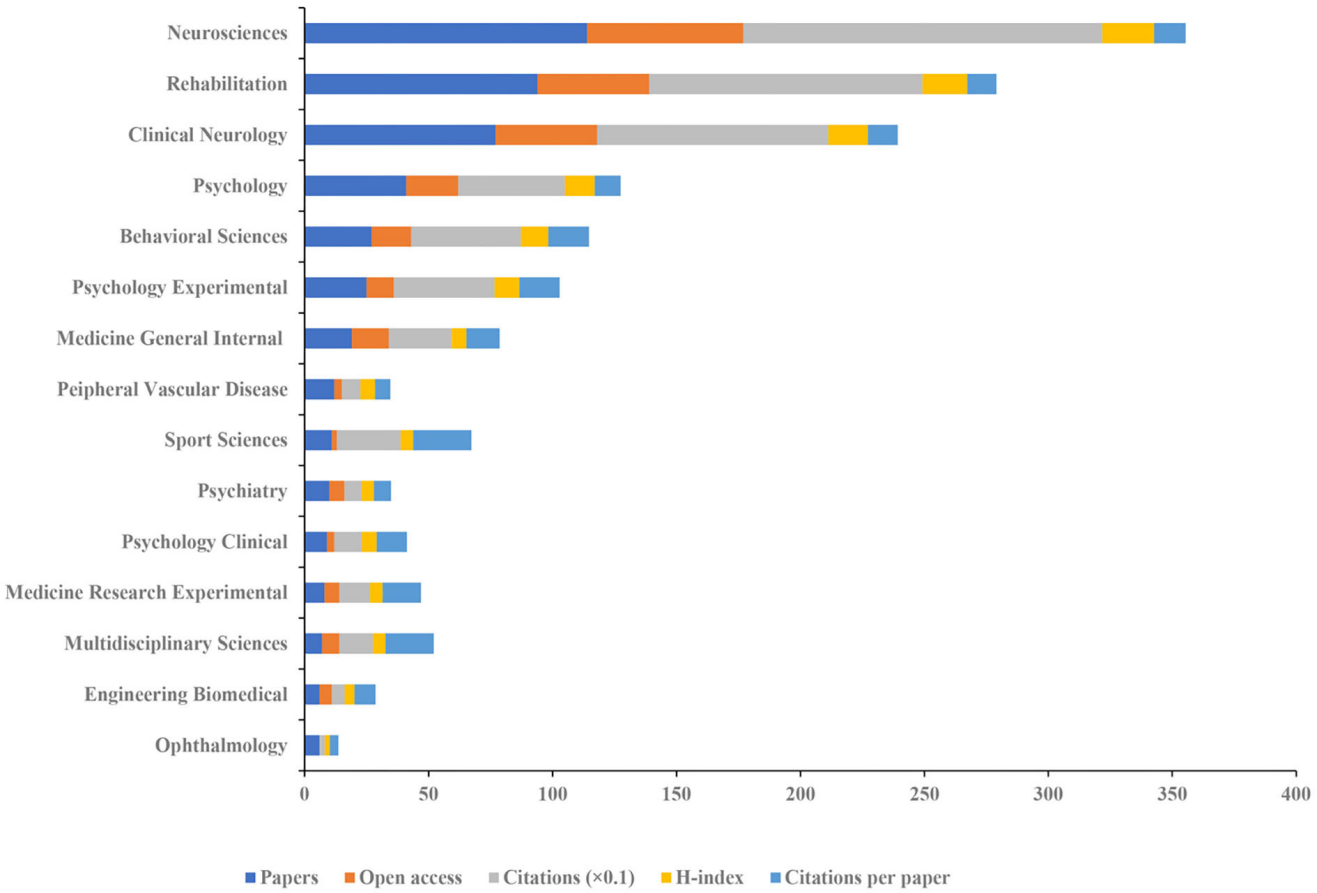
The number of articles, open-access articles, citations, H-index, and citations per paper of the top 15 subject categories of Web of Science.

Among the 43 subject categories, we analyzed the top 15 published disciplines (Figure 5). *Neurosciences* ranked the largest number of publications (*n* = 114), open-access value (*n* = 63), citations (*n* = 1147), and H-index (*n* = 21). *Sport Sciences* had the highest number of citations per paper (*n* = 23.45).

### References analysis

**Figure 6:**
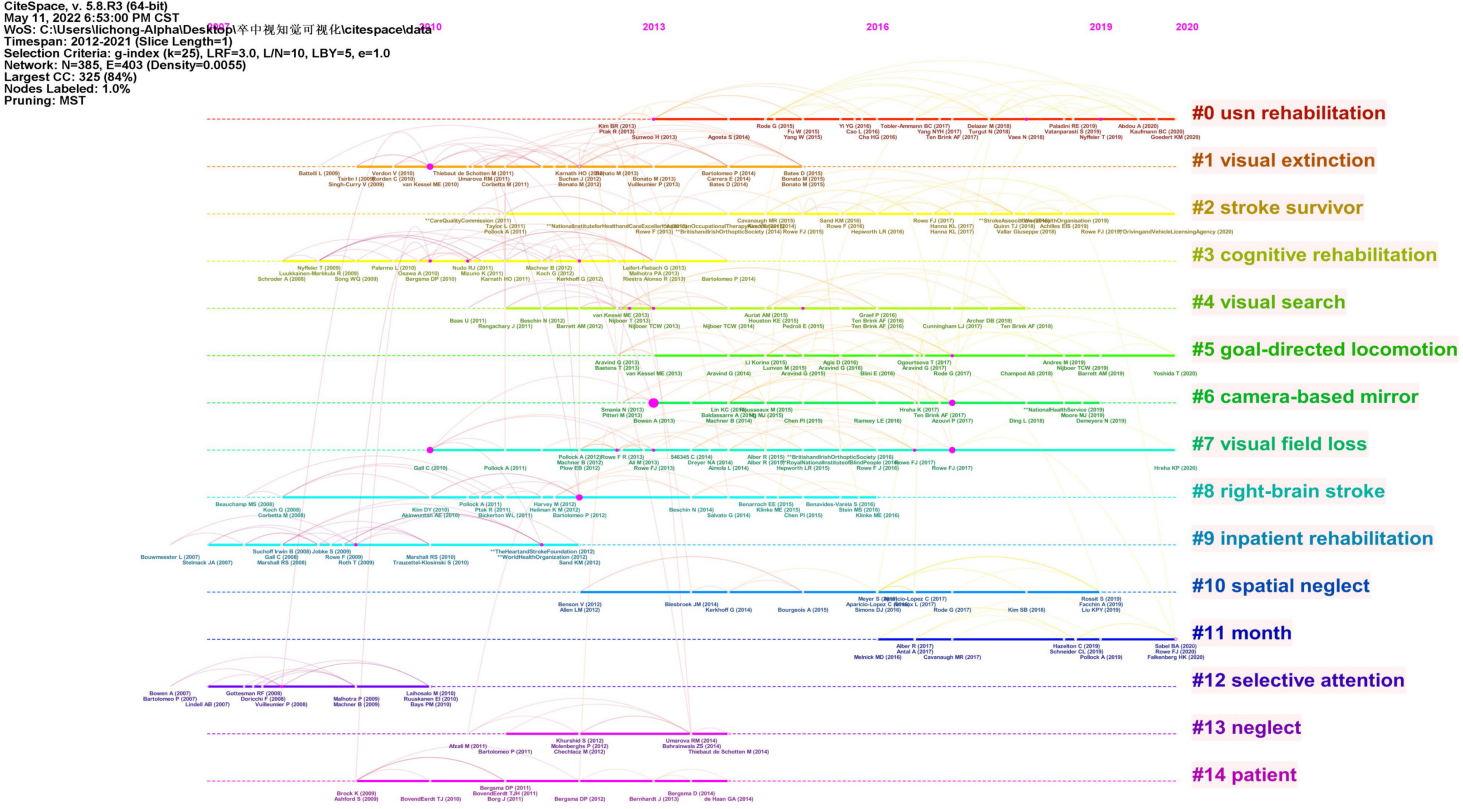
A timeline view of reference co-citation analysis.

Figure 6 shows the timeline view of references in the included 298 papers. The clusters were decided based on keywords and the abstract of included studies. The clustered research categories of reference co-citation analysis were divided into 15 groups (#0–14). The largest cluster (#0) has 32 members which are labeled as *usn rehabilitation*. The most relevant citer to the cluster is “Non-pharmacological interventions for spatial neglect or inattention following stroke and other non-progressive brain injuries” (12). The second-largest cluster (#1) labeled as *visual extinction* has 30 members. The most relevant citer to the cluster is “Multi-tasking uncovers right spatial neglect and extinction in chronic left-hemisphere stroke patients” (18).

### Authoritative countries, institutions, and authors analysis

**Figure 7:**
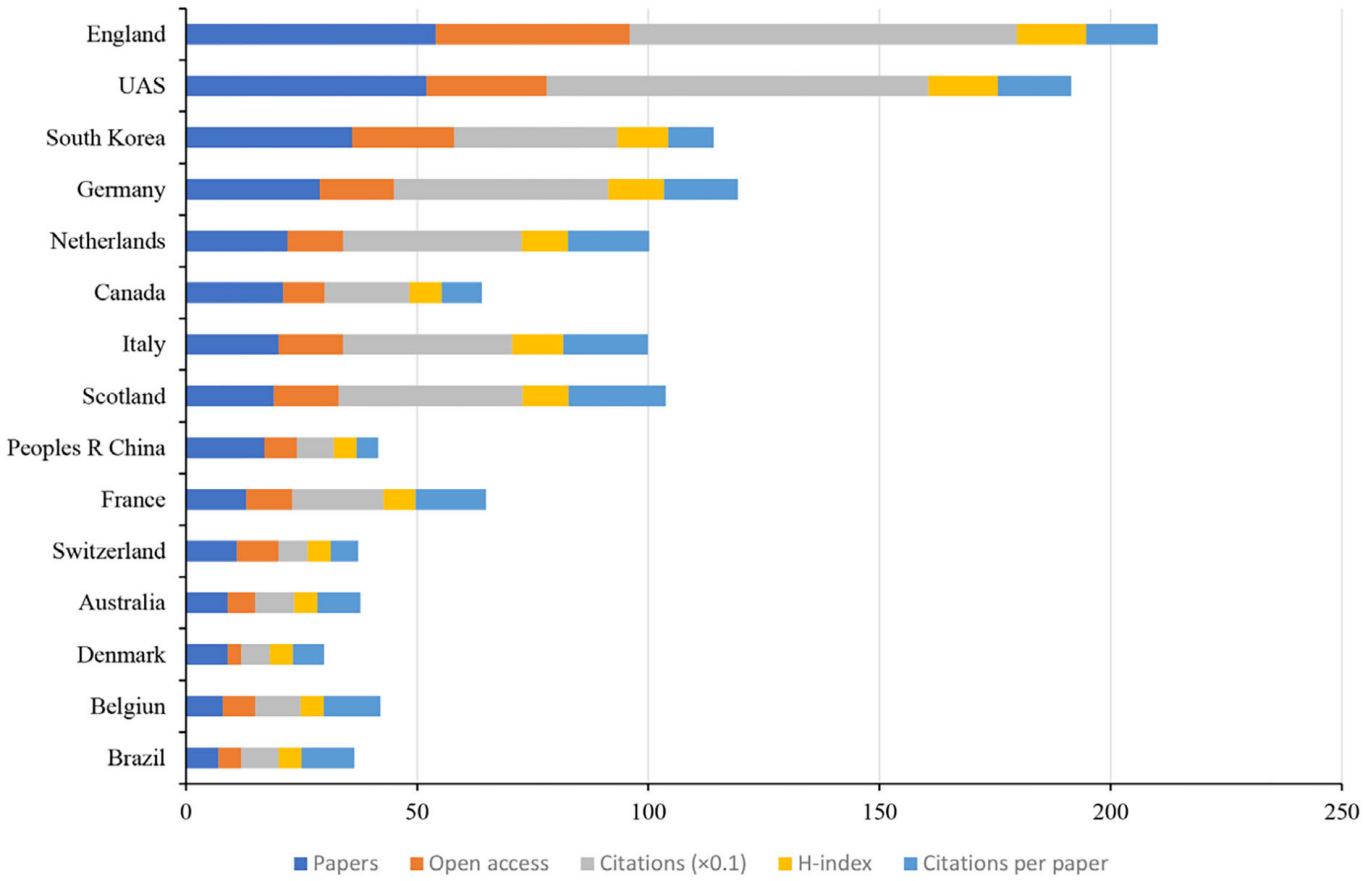
The number of articles, open-access papers, citations, H-index, and citations per paper of the top 15 countries.

**Figure 8:**
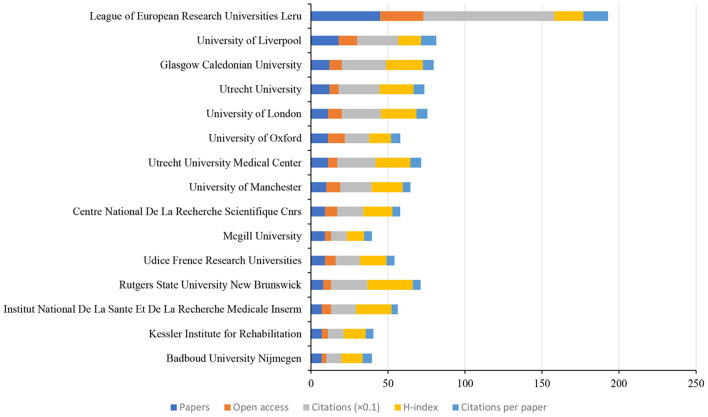
The number of articles, open-access papers, citations, H-index, and citations per paper of the top 15 institutions.

In total, 34 countries, 586 institutions, and 1,182 authors contributed to the related research. The top 15 countries based on the number of publications were shown in Figure 7. England had the greatest number of papers (*n* = 54), open-access value (*n* = 42), citations (*n* = 837), and H-index (*n* = 15). Ranked the highest number of citations per paper (*n* = 20.95). CiteSpace uses “Affiliation” of included studies to analyze authoritative institutions. Each affiliation represents an institution. Figure 8 shows the top 15 institutions in terms of the number of papers. The highest amounts of publications (*n* = 45), open-access value (*n* = 28), citations (*n* = 850), and citations per paper (*n* = 16) were reported at the League of European Research Universities Leru. The top 15 authors and co-cited authors were ranked based on the number of papers published (Table 2). Rowe FJ ranked first (*n* = 17) in terms of publications published, followed by Hepworth LR (*n* = 10) and Pollock A (*n* = 10).

**Table 2:** The top 10 authors, co-cited authors, and co-cited references.

**Author**	**Papers**	**Co-cited author**	**Cited times**	**Co-cited reference**	**Cited times**
Rowe FJ	17	Wilson B	69	Hepworth LR, 2016, Ophthalmology RES IN, 5, 1	18
Hepworth LR	10	Heilman KM	67	Rowe FJ, 2019, PLoS ONE, 14, 0 DOI 10.1371/journal.pone.0213035	13
Pollock A	10	Karnath HO	66	Rowe FJ, 2017, Brain Behav, 7, 0 DOI 10.1002/brb3.778	12
Hazelton C	9	Azouvi P	65	Nijboer TCW, 2013, Cortex, 49, 2021 DOI 10.1016/j.cortex.2012.11.006	10
Nijboer TCW	9	Bowen A	63	Azouvi P, 2017, Ann Phys Rehabil Med, 60, 191 DOI 10.1016/j.rehab.2016.10.006	10
Howard C	8	Halligan PW	55	Karnath HO, 2011, Brain, 134, 903 DOI 10.1093/brain/awq355	9
Lamontagne A	8	Buxbaum LJ	55	Rode G, 2017, Ann Phys Rehabil Med, 60, 177 DOI 10.1016/j.rehab.2016.03.003	9
Demeyere N	6	Corbetta M	55	Bowen A, 2013, Cochrane Db Syst Rev, 0, 0 DOI 10.1002/14651858.CD003586.pub3	8
Ogourtsova T	6	Kerkhoff G	55	Corbetta M, 2011, Annu Rev Neurosci, 34, 569 DOI 10.1146/annurev-neuro-061010-113731	8
Archambault PS	5	Jehkonen M	54	Rowe FJ, 2013, Biomed Res Int, 2013, 0 DOI 10.1155/2013/719096	8

**Figure 9:**
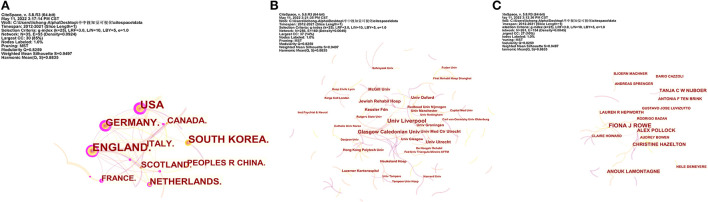
The cooperative network analysis of countries, institutions, and authors. **(A)** Network map of countries. **(B)** Network map of institutions. **(C)** Network map of authors. A link indicates the presence of co-authorship or a co-occurrence relationship.

Figure 9 shows the collaboration maps in different countries, institutions, and authors. In terms of centrality, the top three countries are the USA (*n* = 0.48), Germany (*n* = 0.44), and England (*n* = 0.39). The top three centrality institutions are Glasgow Caledonian Univ (*n* = 0.03), Univ Glasgow (*n* = 0.02), and Univ Strathclyde (*n* = 0.02). The top three centrality authors are Fiona J Rowe (*n* = 0.01), Christine Hazelton (*n* = 0.01), and Verity Longley (*n* = 0.01).

### Keywords analysis

**Figure 10:**
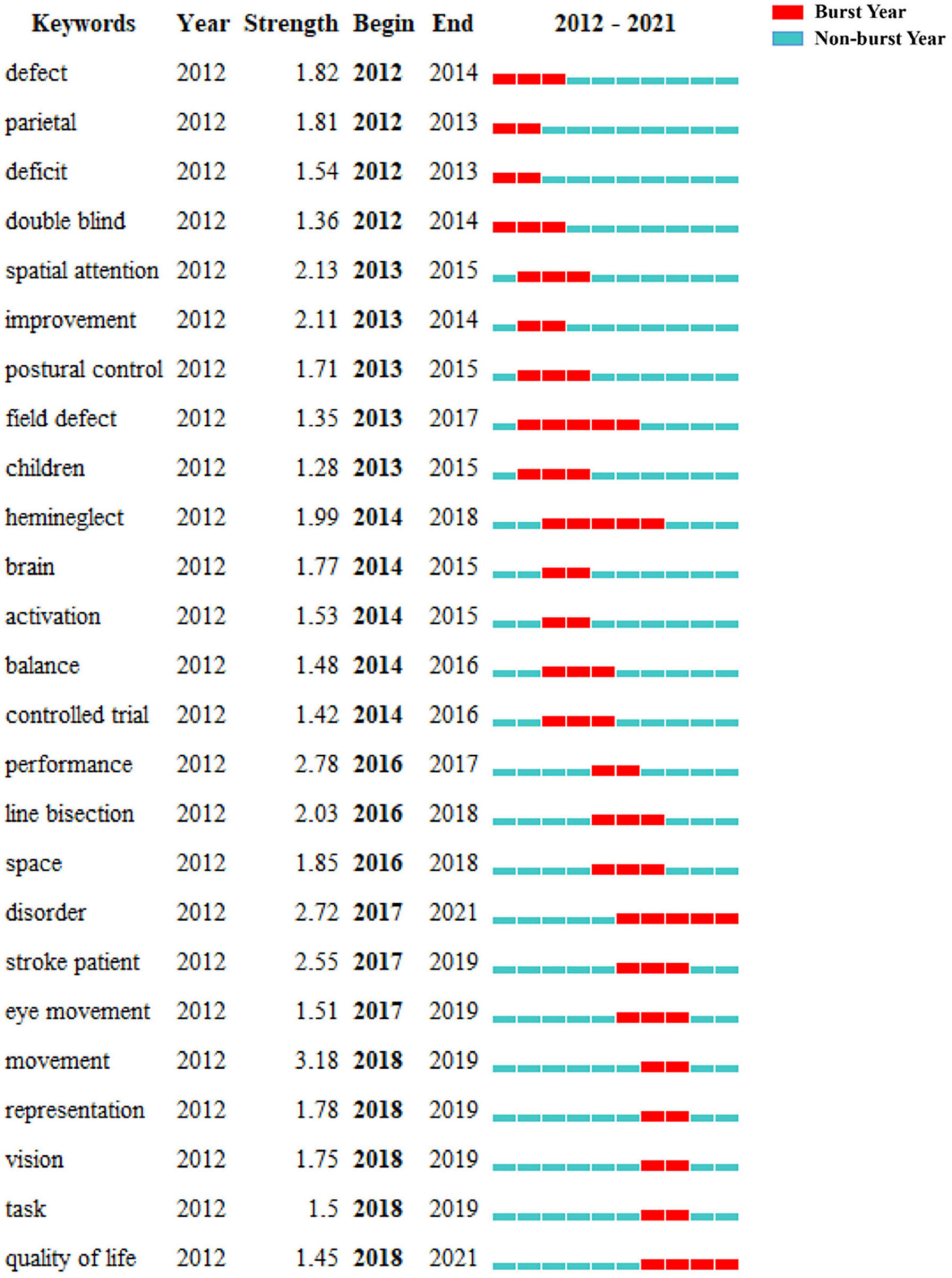
The top 25 keywords with the strongest citation bursts.

Figure 10 shows the top 25 keywords with the strongest citation bursts. The keyword with the highest burst value is movement (*n* = 3.18), and the keywords with the longest burst period are *field defect, hemineglect, disorder*, and *quality of life*. By the end of 2021, the keywords with the most outbreaks of cited publications included *disorder* (2017–2021) and *quality of life* (2018–2021).

### Features of the 10 most frequently cited papers

The production table of the top 10 most frequently cited papers is shown in Table 3. The top 10 papers are cited 888 times, accounting for 24.76% of the total cited numbers (*n* = 3,586). The most cited paper (143 citations) by Bowen et al. with the title “Cognitive rehabilitation for spatial neglect following stroke Evidence-Based Cognitive Rehabilitation: Systematic Review of the Literature From 2009 Through 2014” was published in 2013 in the Cochrane Database of Systematic Reviews (19). Two of the top 10 papers were published in journals with IF ≥ 10 (*Brain* and *Nature Reviews Neurology*).

**Table 3:** The top 10 articles with the most citation frequency.

**Title**	**First author**	**Journal**	**Impact Factor**	**Year**	**Citation (WoS)**	**WoS categories**	**Category ranking**
Cognitive rehabilitation for spatial neglect following stroke	Bowen	Cochrane Database of Systematic Reviews	9.289	2013	143	Medicine, General & Internal	11/169
Evidence-Based Cognitive Rehabilitation: Systematic Review of the Literature From 2009 Through 2014	Cicerone	Archives of Physical Medicine and Rehabilitation	3.966	2019	135	Rehabilitation; Sport Sciences	5/68; 23/88
White matter lesional predictors of chronic visual neglect: a longitudinal study	Lunven	Brain	13.501	2015	118	Clinical Neurology; Neurosciences	6/208; 10/273
Time course of visuospatial neglect early after stroke: A longitudinal cohort study	Nijboer	Cortex	4.027	2013	103	Behavioral Sciences; Neurosciences	7/53; 114/273
Stroke vision, aphasia, neglect (VAN) assessment novel emergent large vessel occlusion screening tool: pilot study and comparison with current clinical severity indices	Teleb	Journal of Neurointerventional Surgery	5.836	2017	76	Neuroimaging; Surgery	2/14; 16/211
The Impact of Recovery of Visuo-Spatial Neglect on Motor Recovery of the Upper Paretic Limb after Stroke	Nijboer	PLoS ONE	3.24	2014	75	Multidisciplinary Sciences	26/72
Anosognosia, neglect, extinction and lesion site predict impairment of daily living after right-hemispheric stroke	Vessel	Cortex	4.027	2013	63	Behavioral Sciences; Neurosciences	7/53; 114/273
A Prospective Profile of Visual Field Loss following Stroke: Prevalence, Type, Rehabilitation, and Outcome	Rowe	Biomed Research International	3.411	2013	62	Biotechnology & Applied Microbiology; Medicine, Research & Experimental	70/159; 80/140
Prism adaptation for spatial neglect after stroke: translational practice gaps	Barrett	Nature Reviews Neurology	42.937	2012	57	Clinical Neurology	2/208
Comparison of Visual Field Training for Hemianopia With Active vs. Sham Transcranial Direct Cortical Stimulation	Plow	Neurorehabilitation and Neural Repair	3.919	2012	56	Clinical Neurology; Rehabilitation	76/208; 6/68

WoS, Web of Science.

## Discussion

### Global research trends on the role of visual perception after stroke

In this study, we performed a bibliometric analysis of studies focused on the role of visual perception after stroke in the past 10 years. The results show that the number of publications represents a continuous but unstable growth trend yearly, with the most obvious growth trend from 2016 to 2021. In addition, the amount of citations shows a continuous growth yearly from 8 to 774. These results indicate that studies focus on the role of visual perception after stroke attracting more and more attention from all over the world.

In terms of authoritative journals, *Journal of Physical Therapy Science* (*n* = 14), *Neurorehabilitation and Neural Repair* (*n* = 14), and *Topics in Stroke Rehabilitation* (*n* = 11) ranked the top three. Among the top 15 journals, only three journals were Q1. In addition, none of the top 15 journals had an IF of more than 5. These results indicate that the quality of studies focused on the role of visual perception after a stroke still needs to strengthen.

In the field of authoritative countries, England (*n* = 54), the USA (*n* = 52), and South Korea (*n* = 36) are the major countries contributing to research on the role of visual perception after stroke. These three countries published 142 papers, accounting for 47.65% of the total number of articles included in this study. In terms of authoritative institutions, the League of European Research Universities ranked first (*n* = 45) based on the number of publications, which is a consortium of leading research universities in Europe. In addition, this institution also had the highest open-access value (*n* = 28), citations (*n* = 850), and citations per paper (*n* = 16). In terms of authoritative authors, Rowe FJ is the most influential author, with the highest volume of articles (*n* = 17), open-access value (*n* = 12), and H-index (*n* = 9). In addition, this author also demonstrated the highest centrality (0.01). From the perspective of the cooperative network, the USA had the greatest centrality (0.48), followed by Germany (0.44) and England (0.39). Glasgow Caledonian Univ is the top institution with the highest centrality (0.03), followed by Univ Glasgow (0.02) and Univ Strathclyde (0.02). It is worth noting that the countries and institutions with the largest volume of publications do not have the highest intermediary centrality. These results indicate that future studies involved in the role of visual perception after stroke should strengthen the cooperation between different countries and institutions.

### Research hotspots on role of visual perception after stroke

Among all the discipline categories of WoS, *Neurosciences, Rehabilitation, Clinical Neurology, Psychology*, and *Behavioral Sciences* are mainly concentrated, indicating that the study on the role of visual perception after stroke focuses on rehabilitation from the perspective of clinical neurology. In addition, reference analysis showed that the most relevant citer to the biggest cluster is “Non-pharmacological interventions for spatial neglect or inattention following stroke and other non-progressive brain injuries.” This review indicated that no rehabilitation approach can be supported or refuted for spatial neglect based on the current evidence from RCTs (12). Future studies need to carry out high-quality research on non-pharmacological interventions for spatial neglect after stroke.

The evolution of a knowledge domain can be reflected by keywords. In terms of count numbers based on keyword analysis, *rehabilitation* (*n* = 85) ranked first, followed by *hemispatial neglect* (*n* = 72) and *recovery* (*n* = 66). In terms of the top 25 keywords with the strongest citation bursts, the keywords with the longest burst period are *field defect, hemineglect, disorder*, and *quality of life*. These four terms indicate potential research hotspots. We give a detailed description as follows:

#### Field defect

Visual field defect is not a visual perceptual defect but can coexist. A study indicated that visual field defects (VFD) affect nearly 30% of patients with unilateral post-chiasmal brain damage (20). The study indicated that having a visual field defect after ischemic stroke is independently associated with increased mortality (21). A systematic review showed that an exception involving compensational strategies may have positive effects on VFD (22). In addition, the Cochrane database systematic review indicated that compensatory scanning training may be more beneficial for VFD (23). However, the evidence for the effect of interventions for VFD is mostly of very low quality, and the effects are uncertain (23–25). Therefore, further studies with high-quality methodology and large sample sizes involving stroke patients with VFD are needed.

#### Hemineglect

Hemineglect is a visual cognitive impairment typically occurring after damage to the parietal cortex, affecting 23% of stroke patients (26). In stroke patients, hemineglect produces a lower capacity for rehabilitation (27). The Cochrane database systematic review indicated that no rehabilitation approach can be supported or refuted based on current evidence from randomized controlled trials for hemineglect after stroke (12). However, a review suggested the beneficial effect of repetitive transcranial magnetic stimulation (rTMS) with moderate quality evidence for hemineglect (28). In addition, one study indicated that interaction patterns mediated by white matter tracts linking cortical nodes of attention-oriented networks, consolidated by further studies, may help develop and customize brain stimulation approaches for the rehabilitation of hemineglect (29). Therefore, future studies should focus on potential non-invasive brain stimulation paradigms for hemineglect after stroke.

#### Disorder

Visual perceptual deficits include apperceptive and associative agnosia, prosopagnosia, akinetopsia, and achromatopsia. Rowe et al. reported that 20.5% of stroke patients with a suspected visual difficulty have visual perceptual deficits (30). At present, various tools are available to screen for post-stroke visual impairment, such as *Vision in Stroke Standardized Screening Form* (30), *Checklist for Vision Problems Post-stroke* (31) et al. However, current tools employ non-standardized assessments and rarely cover higher visual perceptual deficits after stroke (11). In addition, most tools cannot be used for patients with aphasia or communicative deficits (32). Furthermore, these tools are not specific to visual perception disorders after stroke. Therefore, future recommended research should develop a single standardized comprehensive tool for visual perceptual impairments after stroke.

#### Quality of life

From the perspective of functional levels, visual perceptual impairments can significantly reduce the quality of life after stroke, such as being unable to live independently, or return to work (33, 34). Identifying visual perceptual impairment after stroke can strengthen rehabilitation and improve the quality of life for these patients (32). Existing studies have proved that the rehabilitation of visual field loss after stroke is mainly from the point of view of restitution, compensation, and substitution (35, 36). Whether these ways can be used to treat visual perceptual deficits after stroke is unclear. Therefore, multicenter, large sample clinical trials are needed to explore potential treatments from these three ways to improve the quality of life for patients with visual perceptual impairment in the future.

### Future direction on role of visual perception after stroke

According to this bibliometric study, future studies on visual perception after stroke may be carried out from the following aspects. First, basic research is needed to explore the underlying mechanism of different conditions of visual perceptual impairments after stroke. In addition, it is necessary to develop a simple and comprehensive scale for the screening of visual perceptual impairments after stroke. Furthermore, multi-center and large-sample clinical trials are needed to verify potential efficacy treatments for visual perceptual impairments after stroke.

## Strengths and limitations

To the best of our knowledge, this study is the first to use CiteSpace software to perform a bibliometric analysis of publications on the role of visual perception after stroke in the last decade. However, this research also has certain limitations. We only analyzed publications in the WoS database due to a limitation of the CiteSpace software. In addition, this study lacked an assessment of the overall quality of included studies. Additionally, not all types of visual perceptual defects were used as keywords for search strategy.

## Conclusion

This study analyzes the papers on the role of visual perception after stroke in the past 10 years and provides a new perspective for research in this field. The most influential author, institution, journal, and country were Rowe FJ, League of European Research Universities, *Journal of Physical Therapy Science*, and England, respectively. The keywords analysis indicated that current studies related to visual perception after stroke focus on the impact, assessment, and rehabilitation of visual perceptual impairment. In the future, large sample and randomized controlled trials are needed to carry out to identify potential screening tools and efficacy treatments for visual perceptual impairments after stroke.

## Data availability statement

The raw data supporting the conclusions of this study are included in the article/Supplementary material.

## Author contributions

JN and ZW contributed to the conception of the study. NZ and CL performed the data analyses and wrote the manuscript. JC and XL revised the manuscript. All authors contributed to the article and approved the submitted version.

## Funding

This work was supported by the Startup Fund for Scientific Research of Fujian Medical University (No. 2019QH1100).

## Conflict of interest

The authors declare that the research was conducted in the absence of any commercial or financial relationships that could be construed as a potential conflict of interest.

## Publisher's note

All claims expressed in this article are solely those of the authors and do not necessarily represent those of their affiliated organizations, or those of the publisher, the editors and the reviewers. Any product that may be evaluated in this article, or claim that may be made by its manufacturer, is not guaranteed or endorsed by the publisher.

## References

[1] Cavanagh P. Visual cognition. Vision Res. (2011) 51:1538–51. 10.1016/j.visres.2011.01.015 21329719 PMC3204942

[2] Dosher B Lu ZL. Visual perceptual learning and models. Annu Rev Vis Sci. (2017) 3:343–63. 10.1146/annurev-vision-102016-061249 28723311 PMC6691499

[3] Rowe FJ Hepworth LR Howard C Hanna KL Currie J. Impact of visual impairment following stroke (IVIS study): a prospective clinical profile of central and peripheral visual deficits, eye movement abnormalities and visual perceptual deficits. Disabil Rehabil. (2020) 13:1–15. 10.1080/09638288.2020.1859631 33347793

[4] Rowe FJ Hepworth LR Howard C Hanna KL Cheyne CP Currie J. High incidence and prevalence of visual problems after acute stroke: An epidemiology study with implications for service delivery. PLoS ONE. (2019) 14:e213035. 10.1371/journal.pone.0213035 30840662 PMC6402759

[5] Rowe F Brand D Jackson CA Price A Walker L Harrison S. Visual impairment following stroke: do stroke patients require vision assessment? Age Ageing. (2009) 38:188–93. 10.1093/ageing/afn230 19029069

[6] Siong KH Woo GC Chan DYL Chung KYK Li LSW Cheung HKY. Prevalence of visual problems among stroke survivors in Hong Kong Chinese. Clin Exp Optometry. (2014) 97:433–41. 10.1111/cxo.12166 25138748

[7] Colwell MJ Demeyere N Vancleef K. Visual perceptual deficit screening in stroke survivors: evaluation of current practice in the United Kingdom and Republic of Ireland. Disabil Rehabil. (2021) 1–13. 10.1080/09638288.2021.1970246 34455876

[8] Jehkonen M Ahonen JP Dastidar P Koivisto AM Laippala P Vilkki J. Visual neglect as a predictor of functional outcome one year after stroke. Acta Neurol Scand. (2000) 101:195–201. 10.1034/j.1600-0404.2000.101003195.x 10705943

[9] Mercier L Audet T Hébert R Rochette A Dubois MF. Impact of motor, cognitive, and perceptual disorders on ability to perform activities of daily living after stroke. Stroke. (2001) 32:2602–8. 10.1161/hs1101.098154 11692024

[10] Sand KM Wilhelmsen G Naess H Midelfart A Thomassen L Hoff JM. Vision problems in ischaemic stroke patients: effects on life quality and disability. Eur J Neurol. (2016) 231:1–7. 10.1111/ene.12848 26563092

[11] Vancleef K Colwell MJ Hewitt O Demeyere N. Current practice and challenges in screening for visual perception deficits after stroke: a qualitative study. Disabil Rehabil. (2020) 10:1–10. 10.1101/19013243 33016779

[12] Longley V Hazelton C Heal C Pollock A Woodward-Nutt K Mitchell C. Non-pharmacological interventions for spatial neglect or inattention following stroke and other non-progressive brain injury. Cochr Database Syst Rev. (2021) 7:CD003586. 10.1002/14651858.CD003586.pub4 34196963 PMC8247630

[13] Chen C. Searching for intellectual turning points: progressive knowledge domain visualization. Proc Natl Acad Sci USA. (2004) 101 (Suppl. 1):5303–10. 10.1073/pnas.0307513100 14724295 PMC387312

[14] Chen C Hu Z Liu S Tseng H. Emerging trends in regenerative medicine: a scientometric analysis in CiteSpace. Expert Opin Biol Ther. (2012) 12:593–608. 10.1517/14712598.2012.674507 22443895

[15] Chen C Dubin R Kim MC. Emerging trends and new developments in regenerative medicine: a scientometric update (2000 - 2014). Expert Opin Biol Ther. (2014) 14:1295–317. 10.1517/14712598.2014.920813 25077605

[16] Li C Shu X Liu X. Research hotspots and frontiers in post stroke pain: a bibliometric analysis study. Front Mol Neurosci. (2022) 15:905679. 10.3389/fnmol.2022.905679 35645732 PMC9137410

[17] Yin M Wang H Sun Y Xu C Ye J Ma J. Global trends of researches on lumbar spinal stenosis: a bibliometric and visualization study. Clin Spine Surg. (2022) 35:E259–66. 10.1097/BSD.0000000000001160 33769984

[18] Blini E Romeo Z Spironelli C Pitteri M Meneghello F Bonato M. Multi-tasking uncovers right spatial neglect and extinction in chronic left-hemisphere stroke patients. Neuropsychologia. (2016) 92:147–57. 10.1016/j.neuropsychologia.2016.02.028 26948071

[19] Bowen A Hazelton C Pollock A Lincoln NB. Cognitive rehabilitation for spatial neglect following stroke. Cochr Database Syst Rev. (2013) 7:CD003586. 10.1002/14651858.CD003586.pub3 23813503 PMC6464849

[20] Zhang X Kedar S Lynn MJ Newman NJ Biousse V. Homonymous hemianopia in stroke. J Neuroophthalmol. (2006) 26:180–3. 10.1097/01.wno.0000235587.41040.39 16966935

[21] Sand KM Naess H Thomassen L Hoff JM. Visual field defect after ischemic stroke-impact on mortality. Acta Neurol Scand. (2018) 137:293–8. 10.1111/ane.12870 29148038

[22] Smedslund G Myrhaug HT. Interventions for Visual Field Defects After Stroke: A Systematic Review. Oslo: Knowledge Centre for the Health Services at The Norwegian Institute of Public Health (NIPH) (2017). 29553682

[23] Pollock A Hazelton C Rowe FJ Jonuscheit S Kernohan A Angilley J. Interventions for visual field defects in people with stroke. Cochrane Database Syst Rev. (2019) 5:D8388. 10.1002/14651858.CD008388.pub3 31120142 PMC6532331

[24] Luu S Lee AW Daly A Chen CS. Visual field defects after stroke–a practical guide for GPs. Aust Fam Phys. (2010) 39:499–503. 20628665

[25] Alwashmi K Meyer G Rowe FJ. Audio-visual stimulation for visual compensatory functions in stroke survivors with visual field defect: a systematic review. Neurol Sci. (2022) 43:2299–321. 10.1007/s10072-022-05926-y 35149925 PMC8918177

[26] Pedersen PM Jørgensen HS Nakayama H Raaschou HO Olsen TS. Hemineglect in acute stroke–incidence and prognostic implications. The Copenhagen Stroke Study. Am J Phys Med Rehabil. (1997) 76:122–7. 10.1097/00002060-199703000-00007 9129518

[27] Nijboer T van de Port I Schepers V Post M Visser-Meily A. Predicting functional outcome after stroke: the influence of neglect on basic activities in daily living. Front Hum Neurosci. (2013) 7:182. 10.3389/fnhum.2013.00182 23675336 PMC3650314

[28] Kim W Rosselin C Amatya B Hafezi P Khan F. Repetitive transcranial magnetic stimulation for management of post-stroke. J Rehabil Med. (2020) 52:jrm00015. 10.2340/16501977-2637 31922207

[29] Toba MN Zavaglia M Malherbe C Moreau T Rastelli F Kaglik A. Game theoretical mapping of white matter contributions to visuospatial attention in stroke patients with hemineglect. Hum Brain Mapp. (2020) 41:2926–50. 10.1002/hbm.24987 32243676 PMC7336155

[30] Rowe FJ. Accuracy of referrals for visual assessment in a stroke population. Eye. (2011) 25:161–7. 10.1038/eye.2010.173 21127506 PMC3169216

[31] Jolly N Macfarlane A Heard R. Towards gaining the best information about vision to assist the recovery of a patient with stroke. Strabismus. (2013) 2:145–9. 10.3109/09273972.2013.787633 23713940

[32] Hanna KL Hepworth LR Rowe F. Screening methods for post-stroke visual impairment: a systematic review. Disabil Rehabil. (2017) 39:2531–43. 10.1080/09638288.2016.1231846 27669628

[33] Ogourtsova T Archambault PS Lamontagne A. Visual perceptual deficits and their contribution to walking dysfunction in individuals with post-stroke visual neglect. Neuropsychol Rehabil. (2020) 30:207–32. 10.1080/09602011.2018.1454328 29614914

[34] Moore MJ Vancleef K Riddoch MJ Gillebert CR Demeyere N. Recovery of visuospatial neglect subtypes and relationship to functional outcome six months after stroke. Neurorehabil Neural Repair. (2021) 35:823–35. 10.1177/15459683211032977 34269128 PMC8414826

[35] Mansouri B Roznik M Rizzo JR Prasad S. Rehabilitation of visual loss: where we are and where we need to be. J Neuroophthalmol. (2018) 38:223–9. 10.1097/WNO.0000000000000594 29252689

[36] Simpson-Jones ME Hunt AW. Vision rehabilitation interventions following mild traumatic brain injury: a scoping review. Disabil Rehabil. (2019) 41:2206–22. 10.1080/09638288.2018.1460407 29631511

