# Supplementary material for: Research hotspots and frontiers about role of visual perception in stroke: A bibliometric study
Source: Front Neurol. 2022 Sep 16;13:958875. doi: 10.3389/fneur.2022.958875 (PMC9524359; doi:10.3389/fneur.2022.958875)
Supplement: Supplementary file 2 [file Data_Sheet_2.DOC]

MeSh term A: ‘visual perception’ or ‘visual cognition’ or ‘visual memory’ or ‘visuospatial disturbance’ or ‘visual neglect’ or ‘agnosia’ or ‘prosopagnosia’ or ‘akinetopsia’ or ‘achromatopsia’ or ‘hallucination’ or ‘visual*’ or ‘vision*’

MeSh term B: stroke

Search Strategy: ‘Mesh term A’ And ‘Mesh term B’
